# 肺癌术中淋巴结清扫的研究进展

**DOI:** 10.3779/j.issn.1009-3419.2010.07.15

**Published:** 2010-07-20

**Affiliations:** 200030 上海，上海市胸科医院/上海市肺部肿瘤临床医学中心胸外科 Department of Thoracic Surgery, Shanghai Chest Hospital/Shanghai Lung Tumor Clinical Medical Center, Shanghai 200030, China

**Keywords:** 肺肿瘤, 淋巴结转移, 淋巴结清扫, Lung neoplasms, Lymph node metastasis, Lymphadenectomy

## Abstract

淋巴道转移是肺癌最主要的转移途径，也是影响肺癌患者预后的主要因素之一。目前，淋巴结清扫已成为标准肺癌手术的重要组成部分。但是到目前为止，对于不同病理类型、不同病理分期、不同肺叶的肺癌而言，术中淋巴结的清扫方式及范围仍存在一定的争议。本文拟就目前肺癌手术中淋巴结清扫的临床意义、清扫方式、清扫范围的研究新进展进行综述。

## Abstract (en)

Lymphatic metastasis is the main metastatic route for lung cancer, and is also one of the most important prognostic factors. Therefore, lymph node dissection has been an important procedure in standard lung cancer surgery. But so far, the mode and the extent of lymph node dissection during surgery are controversial for lung cancer in different pathological types, stages, or in different lobes. In this review, we summarize advances on the clinical significance, modes and extents of lymph node dissection during lung cancer surgery.

原发性支气管肺癌（以下简称肺癌）是最常见的肺部原发性恶性肿瘤，占肺部肿瘤的95%以上，其中约75%-80%为非小细胞肺癌（non-small cell lung cancer, NSCLC）。目前，外科手术仍是治疗可切除NSCLC的首选方式，但是其术后长期的生存率仍不容乐观。Pisters等^[1]^报道，约有50%的完全性切除的NSCLC患者因远处转移及其相关并发症而导致治疗失败。淋巴结转移是肺癌最主要的转移扩散途径，也是影响预后的重要因素之一。因此，淋巴结清扫已成为标准肺癌手术的重要组成部分，而具体的清扫方式和范围争议多年，迄今仍未完全达成共识，现就相关研究进展予以综述。

## 1 肺癌淋巴结转移方式及特点

一般而言，肺癌淋巴结转移的方式按淋巴结引流途径存在一定的规律，即按照肺内淋巴结→肺门淋巴结→纵隔淋巴结的顺序发生转移^[2]^。此外，亦有部分肺癌患者存在跳跃式转移，即越过N1组淋巴结而直接转移至N2组淋巴结^[3]^。Watanabe等^[4]^根据肿瘤的原发位置，以气管分叉为界，将纵隔淋巴结分为上下两部分，如肿瘤位于上叶，则上纵隔淋巴结称为区域N2，下纵隔淋巴结称为非区域N2；如果肿瘤位于下叶，下纵隔淋巴结称为区域N2，上纵隔淋巴结称为非区域N2。右肺上叶区域N2包括#1组-#4组淋巴结，左肺上叶区域N2包括#1组-#6组淋巴结，中、下叶区域N2包括#7组-#9组淋巴结。

一般情况下^[5]^，右肺上叶肺癌主要为区域性转移至右上纵隔淋巴结（#4组转移概率较高），非区域N2组转移多为隆突下组淋巴结（#7组）；右肺中、下叶肺癌区域性转移至#7组、#9组淋巴结后（#7组转移概率较高），多发生上纵隔非区域N2转移；左肺上叶上段肺癌多为区域性上纵隔淋巴结转移（#5组转移概率较高），其发生非区域淋巴结转移的概率较右肺上叶更低^[6]^；左肺上叶舌段肺癌隆突下组淋巴结（#7组）转移率较高，部分可跨区域至#8组、#9组；左肺下叶肺癌发生区域性淋巴结转移后（#7组、#9组转移概率较高），多延淋巴引流发生上纵隔非区域淋巴结转移，亦可发生右上纵隔交叉转移。国内报道^[7]^也证实，上叶肺癌主要发生上纵隔淋巴结转移；下叶肺癌及右中叶肺癌则易出现上、下纵隔跳跃式淋巴结转移。

## 2 淋巴结切除方式及定义

肺癌区域淋巴结转移具有重要的分期及预后意义。清除区域淋巴结是肺癌手术不可或缺的重要步骤。但是，到目前为止，对于NSCLC外科手术中淋巴结的清扫范围尚未达成广泛一致。为了进一步规范肺癌的外科手术，欧洲胸外科医师协会^[8]^制定了肺癌淋巴结清扫方式的定义、手术操作规范及切除淋巴结病理检查标准指南。将淋巴结清扫方式分为5类：①选择性淋巴结活检（selected lymph node biopsy）：仅对几个可疑淋巴结进行病理检查以确定N分期，用于肿瘤不能被切除的开胸探查手术。②采样或系统性采样（sampling or systematic sampling）：采样指基于手术前影像学或手术中发现，切取几个有代表性的淋巴结；系统性采样指根据原发肿瘤特点切除预先选定的几站区域淋巴结。③系统性淋巴结清扫（systematic node dissection, SND）：系统性清除解剖标志内包含淋巴结在内的所有纵隔组织。要求最少切除3站纵隔淋巴结，并且其中必须包括隆突下淋巴结。除纵隔淋巴结以外，肺门和肺内淋巴结必须一并切除。④肺叶特异性系统淋巴结清除（lobe-specific systematic node dissection）：根据原发肿瘤所在肺叶的不同，清除特定区域内包含淋巴结在内的纵隔组织。⑤扩大性淋巴结清扫（extended lymph node dissection）：通过胸骨正中切口或颈部切口清除双侧纵隔及颈部淋巴结。

## 3 淋巴结清扫方式的研究进展

### 3.1 选择性淋巴结活检

仅用于肿瘤无法切除的剖胸探查术，活检可疑淋巴结用于确定N分期。但是，随着外科分期方法的发展，已很少应用于临床。目前，纵隔镜检查术被认为是评估肺癌患者纵隔淋巴结状况最准确的手段，可应用于#1组、#2组、#3组、#4组、#5组、#6组、#7组淋巴结，其各类并发症的发生率较低，敏感性及特异性分别高达90%和100%^[9, 10]^。此外，超声内镜引导针吸活检术（endoscopic ultrasonography guided fine-needle aspiration, EUS-FNA）可对纵隔镜较难抵达的淋巴结（#3组、#5组、#8组、#9组）进行评估，对于异常淋巴结其敏感性及特异性可达90%和97%^[11, 12]^。支气管内超声引导针吸活检术（endobronchial ultrasound-guided transbronchial needle aspiration, EBUS-TBNA）可对#1组、#2组、#4组、#7组以及#10组淋巴结进行多次针吸活检，其敏感性及特异性可达89%和100%^[13]^。后两者安全性更高，并发症较少而轻微，但有部分患者因标本量不足而出现假阴性诊断。

### 3.2 淋巴结采样及系统性淋巴结清扫

是目前临床工作中最常用的淋巴结清扫方式。两种术式对于肺癌患者预后的影响目前仍存在争议。不主张做系统性淋巴结清扫的学者^[14, 15]^认为，该术式相对增加了手术风险，使手术时间延长，并发症如心律失常、乳糜胸、喉返神经麻痹等发生率相对增高，对患者损伤大、术后胸腔引流量增加、增加死亡率，并且系统性淋巴结清扫不能明显改善患者预后。但是更多的学者^[16, 17]^主张肺癌术中行系统性淋巴结清扫，该术式可更彻底地切除淋巴结，提高肿瘤分期准确性，更大限度地减轻肿瘤负荷，控制因微转移而导致的局部复发及远处转移。并且认为SND与淋巴结采样相比，术后并发症发生率及死亡率无明显统计学差异^[18]^。Keller等^[19]^将373位N分期为N1、N2期的NSCLC患者随机分为淋巴结采样组及系统性淋巴结清扫组，研究不同清扫方式对Ⅱ期-Ⅲa期NSCLC患者生存率的影响。结果：总体而言，系统性淋巴结清扫组的中位生存期为57.2个月，而淋巴结采样组仅为29.2个月；对于N1期患者，系统性淋巴结清扫组及淋巴结采样组的中位生存期分别为66.4个月和45.2个月；对于N2期患者，中位生存期则分别为38.2个月和22.2个月；三组数据间均存在显著统计学差异。且通过多因素分析证实淋巴结采样是影响预后的危险因子，其相对危险（relative risk, RR）值为1.502。吴一龙等^[20]^采用随机分组的方法，研究了532例临床病理分期为Ⅰ期-Ⅲa期NSCLC患者的淋巴结清扫方式对其长期生存率的影响。结果显示：系统性淋巴结清扫组与淋巴结采样组相比，可提高Ⅰ期-Ⅲa期患者长期生存率。特别是对于Ⅰ期NSCLC，系统性淋巴结清扫可更彻底地清除肿瘤细胞，降低术后复发及远处转移的机率；对于已有纵隔淋巴结转移的N2组患者，虽然已进入全身疾病状态，但是肺叶（全肺）切除术+系统性淋巴结清扫仍可改善其长期生存率。Wright等^[21]^对既往3个相关性研究进行系统性回顾及*meta*分析，结果显示：与肺叶（全肺）+系统性淋巴结采样术相比，肺叶（全肺）+系统性淋巴结清扫可延长NSCLC患者的生存期，降低22%的死亡危险。虽然目前已有较多临床相关性研究报道了肺癌术中行系统性淋巴结清扫优于行系统性淋巴结采样。但是，大多数的随机对照试验（randomized control trial, RCT）无法满足CONSORT（Consolidated Standards of Reporting Trials）声明^[22]^对于RCT的要求，会产生对干预因素的估计偏倚，从而使研究结果可信度下降。因此，肺癌术中系统性淋巴结清扫是否优于系统性采样仍有待进一步研究。

### 3.3 肺叶特异性系统淋巴结清除

虽然肺叶（全肺）+系统性淋巴结清扫被大量临床研究推荐为肺癌的标准根治术，但是肺癌术中是否行系统性淋巴结清扫仍然存在着争议，特别是对于临床病理分期为Ⅰ期的NSCLC。随着研究人员对于肺癌淋巴结转移途径研究的深入，肺叶特异性淋巴结转移的模式已逐渐被认识。根据Asamura及Okada等^[23, 24]^的报道，右肺上叶及左肺上叶上段肺癌倾向于发生上纵隔淋巴结转移，且较少越过肺门及上纵隔淋巴结而发生隆突下淋巴结转移；而左肺舌段及右肺中叶肺癌则倾向发生上纵隔及隆突下淋巴结转移；下叶肺癌则较易于隆突下淋巴结转移后向上纵隔淋巴结转移。因此，有学者^[23, 25-27]^提出，如术中切除的“前哨兵淋巴结”冰冻病理呈阴性，则可行肺叶特异性系统淋巴结清除。具体方案如下：

**Table:** 

基于肺叶特异性淋巴结转移模式的选择性淋巴结清扫策略			
淋巴结清扫范围	原发肿瘤部位		
	右肺上叶 左肺上叶上段	右肺中叶 左肺上叶舌段	右肺下叶 左肺下叶
上纵隔淋巴结	建议清扫	建议清扫	注释1
隆突下淋组巴结	注释2	建议清扫	建议清扫
(#7)			
食管旁（#8)及下肺	无需清扫	无需清扫	建议清扫
韧带组（#9)淋巴结			
注释1:术中肺门及隆突下组淋巴结（#7)冰冻呈阴性，则无需清扫			
注释2:术中肺门及上纵隔淋巴结冰冻呈阴性，则无需清扫			

Yoshimasu及Muraoka等^[28, 29]^通过临床回顾性研究报道，对于临床病理分期为Ⅰ期的NSCLC患者，术中行肺叶特异性系统淋巴结清扫，且术中所切除淋巴结冰冻病理证实为阴性者，其5年生存率与系统性淋巴结清扫组相比无统计学差异，且可明显减少出血及术后心律失常等并发症发生率。虽然目前已有不少临床研究证实了肺叶特异性淋巴结清扫的可行性，但是目前仍有两大问题有待解决：①术前及术中如何排除跳跃性转移；②术中冰冻准确性有待进一步提高（特别是对于淋巴结微转移）。

### 3.4 扩大性淋巴结清扫

1990年，Hata等^[30, 31]^首先提出通过胸骨正中及颈部切口行扩大性淋巴结清扫，其优势在于可最大限度地清扫纵隔及颈部淋巴结，确定N分期。但由于此术式存在创伤较大，术中出血较多，术后并发症发生率高等缺陷，所以无法被广大临床医生所接受。之后，Kuzdzał^[32-34]^在此基础上报道了一种新的扩大性淋巴结清扫方式——经颈扩大性纵隔淋巴结清扫术（transcervical extended mediastinal lymphadenectomy, TEMLA）。该术式采用颈部领式切口及胸骨抬高牵引器，可于直视下切除#1组、#2R组、#2L组、#3a组、#4R组、#4L组淋巴结，并可在电视纵隔镜的辅助下清扫#5组、#6组、#7组淋巴结及部分#8组淋巴结。与原方式相比，TEMLA虽然无法对#9组淋巴结进行清扫，但其具有创伤较小、术中及术后并发症发生率较低等优势。与常规开胸手术相比，其对淋巴结的清扫更为彻底、N分期定性更为准确，但TEMLA手术时间较长，对于病灶的切除仍需通过开胸手术或胸腔镜手术完成，且目前仍无相关性研究报道TEMLA对于NSCLC患者的预后有益。因此，其临床应用价值仍存在争议，需临床相关性研究进一步验证。

## 4 总结与展望

淋巴道转移是肺癌最主要的转移扩散途径，也是影响预后的重要指标之一。因此，淋巴结清扫已成为标准肺癌手术的重要组成部分。虽然，大量的临床研究已证实淋巴结清扫可使部分肺癌患者受益，但是到目前为止，对于不同病理类型、不同病理分期、不同肺叶的肺癌而言，术中淋巴结的清扫方式及范围仍存在一定的争议。相信随着临床研究的进一步深入及分子生物学等新技术逐步应用于肺癌研究，更为人性化、更有目的性、更为高效的淋巴结清扫方式会逐步应用于临床，从而为肺癌患者带来福音。
